# Intraperitoneal Administration of S100A8 Ameliorates Experimental Acute Colitis in Rats

**DOI:** 10.3390/biology13110916

**Published:** 2024-11-11

**Authors:** Kano Matsuo, Masaki Ikemoto, Kohki Okada

**Affiliations:** 1 Graduate School of Health Sciences, Kyoto Tachibana University, Kyoto 607-8175, Japan; 2 Division of Clinical Immunology and Rheumatology, Department of Internal Medicine (IV), Faculty of Osaka Medical College, Osaka 569-8686, Japan; 3 Department of Medical Technology and Sciences, Faculty of Health Sciences, Kyoto Tachibana University, Kyoto 607-8175, Japan

**Keywords:** experimental colitis, lipopolysaccharide, macrophage, S100A8, S100A9, tumor necrosis factor-α

## Abstract

S100A8 is a protein that is abundant in neutrophils and macrophages (MΦ), but its role in inflammation remains unclear. This study aimed to assess the immunological role(s) of S100A8 in acute intestinal inflammation in rats and its role in MΦ. Rat recombinant S100A8 (rr-S100A8, 1.0 mg/kg) was intraperitoneally administered daily to rats with 3% dextran sulfate sodium (DSS) (DSS + A8 group)-induced experimental acute colitis. The histological severity score (6.50 ± 0.51, *p* = 0.038) in the DSS + A8 group rats remained lower than that (9.75 ± 1.48) of the rats without S100A8 (DSS group) administration. The tumor necrosis factor-alpha (TNF-α) production in the colon tissues of the rats in the DSS + A8 group (4.76 ± 0.90 pg/mL/g, *p* = 0.042) was significantly suppressed, compared with that of the DSS group (10.45 ± 2.04 pg/mL/g). To stimulate rat peritoneal MΦ, rr-S100A8, the anti-rat S100A8 antibody, and a lipopolysaccharide (LPS) were used in the in vitro experiments. In the MΦ stimulated with rr-S100A8 for 2 h, the mRNA level of intracellular S100A8 (47.41 ± 24.44, *p* = 0.002) increased in an autocrine manner, whereas that of S100A9 (0.24 ± 0.43, *p* = 0.782) was not significant. The TNF-α mRNA level in the MΦ treated with LPS and the anti-rat S100A8 antibody significantly increased (102.26 ± 18.60, *p* = 0.001) compared to that with LPS alone (16.9 ± 8.56). These results indicate that S100A8 can serve as an anti-inflammatory protein in acute inflammation by negatively regulating S100A9 and TNF-α production through inflammatory signaling pathways in MΦ.

## Simple Summary

This study investigated S100A8’s immunological role in acute intestinal inflammation in rats. The rats that developed colitis after regularly drinking 3% dextran sulfate sodium (DSS), and those that were administered rat recombinant S100A8 (rr-S100A8) in addition to DSS were named the DSS + A8 group. The histological severity scores were lower in the DSS + A8 group compared to the DSS group. TNF-α production in the colon tissues was significantly suppressed in the DSS + A8 group. In vitro experiments showed that rr-S100A8 increased intracellular S100A8 mRNA levels in macrophages. The mRNA level of TNF-α significantly increased in macrophages treated with lipopolysaccharide and the anti-rat S100A8 antibody. S100A8 appeared to function as an anti-inflammatory protein by negatively regulating S100A9 and TNF-α production in macrophages.

## 1. Introduction

S100A8 and S100A9 are S100 family proteins that are normally observed in the cytoplasm of neutrophils, monocytes, and macrophages (MΦ) [1,2]. These two proteins were initially identified in the synovial fluid of patients with rheumatoid arthritis; however, they were subsequently observed in several other inflammatory diseases, such as ulcerative colitis (UC) and psoriasis [1,3,4]. S100A8 and S100A9 likely regulate immune function through multiple intracellular signaling pathways using receptors, such as the Toll-like receptor (TLR)-4 and the receptor for advanced glycation end product (RAGE) in immune cells [5,6]. S100A8 contributes to the production of anti-inflammatory cytokines, such as interleukin (IL)-10 and the transforming growth factor (TGF)-β, whereas S100A9 may be involved in the secretion of inflammatory cytokines, such as IL-1β, IL-6, and tumor necrosis factor (TNF)-α [7]. Heterodimers of S100A8 and S100A9 (S100A8/A9) have been observed in patients with inflammatory diseases [1,4]. Additionally, the functional differences between the two proteins may be because of their three-dimensional structures.

UC is a chronic inflammatory condition in the large colon that causes chronic erosions and ulcerations in the colonic mucosa. Various hypotheses exist regarding the occurrence of UC, including those associated with genetic mutations, such as nucleotide-binding oligomerization domain 2/caspase recruitment domain-containing protein 15 (NOD2/CARD15), immunity-related GTPase M (IRGM), and autophagy-related 16 like 1 (ATG16L1), or environmental factors, such as smoking, specific drugs, and foods, but nothing is definitive [8,9,10]. The prevailing theory states that a complex combination of factors causes the onset and worsening of UC. Inflammation in UC is thought to be aggravated by the production of autoantibodies against antigenic substances that penetrate the mucosa of large colon tissues, thereby triggering an excessive immune response [11]. In the gastrointestinal mucosa, certain cytokines are closely associated with the maintenance of the mucosal environment and regeneration. Specifically, the level of TNF-α produced by activated MΦ in intestinal epithelial cells and lamina propria is strongly correlated with the severity of UC [12]. Additionally, MΦ prevent the excessive immune response caused by intestinal bacteria and/or their components by ingesting them, thereby resulting in the maintenance of intestinal homeostasis [13,14]. Therefore, the initial immune reaction of intestinal MΦ to commensal bacteria and their components may trigger UC. Moreover, the number of neutrophils significantly increases in the blood and colon tissues of patients with UC, where they likely play crucial roles(s), and may be closely associated with UC severity [15]. Additionally, CD4+ helper and regulatory T cells modulate the immune environment in the normal intestinal tract, with the overresponsive nature of CD4+ helper T cells in the intestinal tract of patients with UC [16]. In UC, abnormalities in immune cells (number and/or function), specifically MΦ, likely trigger the initial response, and the removal of these causative leukocytes through leukocytapheresis and granulocytapheresis is considered more effective in UC treatment [17]. Other drugs, such as immunosuppressants, anti-inflammatory drugs, and steroids are prescribed to patients based on UC symptoms [18]. However, the range of drugs available for these treatments is limited, with no recent developments. If UC remains severe despite these medications, surgical therapy, such as the resection of colorectal tissue, may be necessary [18].

The relationship between UC and S100A8/A9 has been extensively discussed. Upon inflammation in the intestinal tract of patients with UC, S100A8/A9 is excessively produced in neutrophils and MΦ, potentially resulting in its secretion in stool samples [19]. Therefore, the measurement of S100A8/A9 concentration in the stool is currently adopted as a specific marker to assess its severity as a specific marker in numerous countries [20]. Additionally, serum S100A8/A9 levels are more reliable than C-reactive protein, which is an existing inflammatory marker [21]. Few studies have described a relationship between S100A9 and UC. The activation of the signal transduction activator transcription 3 (STAT3) pathway in the intestinal epithelial cells of the colon by IL-6 results in the excessive secretion of S100A9 and the deterioration of UC [22]. Moreover, the inhibition of S100A9 expression by an inhibitor (paquinimod) in the colon tissue has been demonstrated to enhance the pathology of dextran sulfate sodium (DSS)-induced experimental UC in rats [23]. However, reports exploring the relationship between UC and S100A8 compared to S100A8/A9 or S100A9 are scarce. When experimental colitis was induced using DSS in transgenic rats with high systemic expression of S100A8 (Tg-S100A8), colitis pathogenesis was alleviated [24]. In contrast, the increased expression of S100A8 in the intestinal tract resulted in helper T cell hyperactivity, thereby exacerbating DSS-induced murine colitis [25]. Therefore, the functional role of S100A8 in UC remains unclear. Further assessments are required to clarify the immunological function of S100A8 in UC and its relationship with S100A9 and S100A8/A9.

In this study, we confirmed the pharmacological effects of S100A8 in UC and intraperitoneally administered recombinant rat S100A8 (rr-S100A8) to rats with DSS-induced experimental colitis. Additionally, the MΦ collected from the abdominal cavity of rats were assessed for the functional role of S100A8. We predicted that the treatment of MΦ via rr-S100A8 could suppress the aberrant activation of the MΦ. In addition, we speculated that the administration of rr-S100A8 to rats would improve the pathogenesis of experimental colitis. Few studies have been found that aim to apply the immunological role of S100A8 in the treatment of UC. This study was realized because we were able to purify a large amount of rr-S100A8, and the results obtained would be quite valuable.

## 2. Materials and Methods

### 2.1. Ethics Statement

All the animal experiments adhered to the Animal Research: Reporting of In Vivo Experiments (ARRIVE) guidelines and were approved by the Animal Care and Use Committee of Kyoto Tachibana University (permission number: 20-03). In this experiment, the concentration of DSS was set to 3% and its regular drinking period to 10 days to avoid the fatal severity of UC and the continuation of pain. The humane endpoint was set at a −20% body weight change, and once this criterion was exceeded, euthanasia via cervical dislocation under anesthesia was planned. After all, none of the rats (*n* = 40) used in this study reached this endpoint. The experimental period was up to 20 days, and the body weight and condition of the rats were monitored every day. Dissection was performed after the intraperitoneal administration of anesthetic mixture (0.375 mg/kg medetomidine + 2.0 mg/kg midazolam + 2.5 mg/kg butorphanol) [26]. After blood was drawn from the rats’ hearts under anesthesia, the hearts were removed, and their deaths were quickly confirmed.

### 2.2. Animals

Wild-type Wistar rats (WT, male, 10-week-old, 220–250 g/rat) were purchased from Shimizu Laboratory Supplies Co., Ltd., Kyoto, Japan. The animals were acclimated for approximately 1 week before the experiment, with free access to regular diets (MF, Oriental Yeast Co., Ltd., Tokyo, Japan) and water. During the experiments, the animals were housed in individual sawdust-lined plastic cages under controlled temperature (22 °C) and humidity (60%) conditions, with day–night cycles regulated using artificial light (12/12 h).

### 2.3. Reagents

DSS salts (molecular weight, 36,000–50,000), diaminobenzidine (DAB) and o-phenylenediamine dihydrochloride (o-PD) tablets, 1% eosin Y solution, and Escherichia coli O111:B4-derived lipopolysaccharide (LPS) were purchased from Wako Pure Chemical Industries, Ltd., Tokyo, Japan. Mayer’s hematoxylin solution was purchased from Muto Pure Chemicals Co., Ltd. (Tokyo, Japan). Monoclonal antibodies against rat S100A8 (Ab2H6 and Ab8A6) and S100A9 (Ab15E9 and Ab10D11) were purchased from Yamasa Co. Ltd. (Chiba, Japan). Anti-S100A8 IgG (anti-A8), anti-S100A9 IgG (anti-A9), PRO-PREP^TM^ Protein Extraction Solution (cell/tissue), anti-neutrophil elastase (NE) IgG, IL-6 PicoKine^TM^, and enzyme-linked immunosorbent assay (ELISA) kits for IL-1β, and TNF-α were purchased from Cosmo Bio Co., Ltd., Tokyo, Japan. Biotin Labeling Kit-SH and Peroxidase (horseradish peroxidase [HRP]) Labeling Kit-SH were purchased from DOJINDO Laboratories (Kumamoto, Japan). An avidin–biotin Blocking Kit, HRP-labeled anti-rabbit IgG (H + L), goat–poly IgG, tetramethylrhodamine (TRITC)-labeled anti-mouse IgG, and fluorescein isothiocyanate (FITC)-labeled streptavidin (STA-FITC) were purchased from Funakoshi Co., Ltd. (Tokyo, Japan). VECTASHIELD Antifade Mounting Medium containing 4′,6-diamidino-2-phenylindole (DAPI) was purchased from Vector Laboratories, Inc. (Newark, CA, USA). Go Taq Green Master Mix for polymerase chain reactions (PCRs) was purchased from Promega Co., Ltd. (Madison, WA, USA). Clinical thioglycolate medium E-MC17 was supplied by Eiken Chemical Co., Ltd. (Tokyo, Japan). The TRIzol reagent, the Maxima H Minus cDNA Synthesis Master Mix, the PowerUp SYBR Green Master Mix for real-time PCR, and all the primers were purchased from Thermo Fisher Scientific (Waltham, MA, USA). The primers used were similar to those used in our previous study and are listed in Table 1 [7]. All the other reagents were purchased from Wakenyaku Co., Ltd. (Kyoto, Japan), Nacalai Tesque Co., Ltd. (Kyoto, Japan), and Bio-Rad Laboratories, Inc. (Hercules, CA, USA).

### 2.4. Preparation and Purification of Recombinant Rat S100A8 and S100A9

In this study, previously prepared rat S100A8 cDNA was used [27,28]. The rr-S100A8 protein was purified as previously described [29,30]. Rat S100A8 cDNA was inserted into the pCold-I vector and transformed into *E. coli* cells, which were cultured at 37 °C for 3–5 h. When absorbance at 600 nm ranged from 0.4 to 0.8, the temperature of the medium was lowered to 15 °C. Subsequently, isopropyl-β-D-galactopyranoside (final concentration of 1 mmol/L) was added to the medium to induce the expression of the target protein, and the cells were further cultured at the same temperature overnight. *E. coli* cells were subsequently harvested through centrifugation (8000 rpm, room temperature) and washed once with phosphate-buffered saline (PBS, pH 7.4). After the ultrasonication of the recombinant cells, the rr-S100A8 was purified using Ni-agarose and diethylethanolamine columns. Sodium dodecyl sulfate–polyacrylamide gel electrophoresis (SDS-PAGE) was performed in the presence and absence of 2-mercaptoethanol (2-ME) to confirm the single band of rr-S100A8, according to the method described by Towbin et al. [31]. The rr-S100A8 protein was mixed with 20% glycerol and stored at 4 °C. Simultaneously, the concentration of endotoxins in the rr-S100A8 was measured using the commercially available ToxinSensor Endotoxin Detection System (GenScript Biotech Corp., Piscataway, NJ, USA). The endotoxin concentration in each sample was <1.0 EU/mg protein. This indicated that the contaminated endotoxin exhibited minimal effect on the activation of the MΦ.

### 2.5. Protocol for Animal Experiments

Before the animal experiments, DSS salt was dissolved in distilled water (DW) at a 3% concentration (3% DSS). In this study, two types of in vivo experiments were conducted over different experimental periods (10 or 20 days).

Animal experiment ①

The rats were treated with 3% DSS for 10 days and subjected to animal experiment ①. This group was further divided into three groups (1)–(3) (*n* = 6 each):

(1) Normal group: WT rats were allowed to drink DW for 10 days. During this period, 50 mM of PBS (pH 7.4) was administered intraperitoneally daily;
(2) DSS group: WT rats were free to consume 3% DSS for 10 days. During this period, 50 mM of PBS was administered intraperitoneally daily;
(3) DSS + A8 group: WT rats were allowed free access to 3% DSS for 10 days. During this period, rr-S100A8 (1.0 mg/kg) was administered intraperitoneally daily.

Animal experiment ②

The rats were treated with 3% DSS for 20 days and subjected to animal experiment ②. This group was further divided into two groups (4) and (5) (*n* = 6 each):

(4) DSS/Normal group: WT rats were free to drink 3% DSS for the first 10 days, followed by DW alone over the last 10 days. During the latter period, 50 mM PBS was administered intraperitoneally daily for 10 days;
(5) DSS/A8 group: WT rats were free to drink 3% DSS for the first 10 days. During the latter period (10 days), they received DW alone, and rr-S100A8 (1.0 mg/kg) was simultaneously administered daily for the last 10 days.

**Figure 1:**
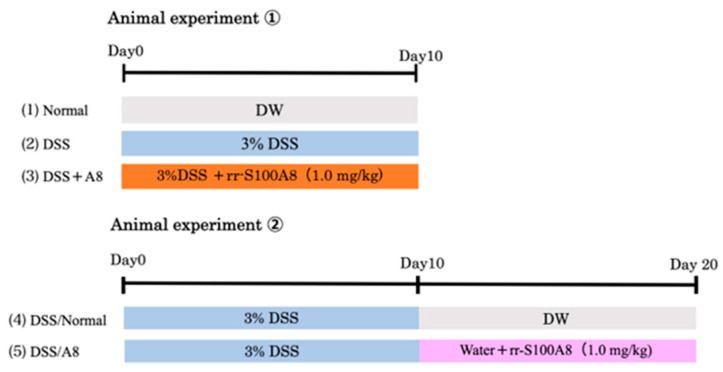
In vivo experimental protocol. Five groups are as follows: (1) Normal; (2) DSS; (3) DSS + A8; (4) DSS/Normal; and (5) DSS/A8 (see Section 2).

Figure 1 demonstrates the strategy for animal experiments in each group (1)–(5). During the experimental period, the body weight of each rat was measured every morning, and disease activity index (DAI) scores were assessed based on the criteria listed in Table 2 [32]. After the experimental period, each rat was euthanized under anesthesia and blood samples (3 mL/rat) were collected through intracardiac puncture. The large colon was quickly removed, and its length was measured. The specimens were fixed in 10% formalin/0.1 M PBS at pH 7.4 for histological assessment and subsequently embedded in paraffin. Total protein and mRNA were extracted from the residual unfixed tissue as described below.

### 2.6. Sample Preparation for the Protein Assay

The large intestine removed from each experimental animal was weighed separately. Subsequently, 300 mg of each sample was incubated in 0.5 mL PRO-PREP^TM^ Protein Extraction Solution (cell/tissue) for 10 min, followed by centrifugation (4 °C, 12,000 rpm, 10 min). The resulting supernatants were transferred into polycarbonate tubes (1.5 mL) and stored at −80 °C.

### 2.7. Sample Preparation for the mRNA Assay

Each tissue (300 mg) was incubated in 1.0 mL TRIzol™ reagent for 10 min, followed by centrifugation (4 °C, 12,000 rpm, 15 min). Total mRNA was extracted from each sample following the manufacturer’s protocol and treated with 8 M LiCl to prevent the effect of DSS on RNA reverse transcription. cDNA was synthesized from the mRNA using the Maxima H Minus cDNA Synthesis Master Mix, following the manufacturer’s protocol.

### 2.8. Microscopic Observation of Rectal Tissues

Severe inflammation in the rectal tissue of each rat was observed under a microscope. The tissue sections (3-µm-thick) were prepared and stained with hematoxylin and eosin (H&E). The tissue damage was assessed using histological severity (HIS) scores; severity was assessed based on H&E staining, and the extent of damage was scored based on established criteria on a scale of 0–14 (Table 3) [33]. The NE expression was detected using anti-NE IgG immunohistochemical (IHC) staining, as previously described [24]. The Ab15E9 was labeled with biotin using the Biotin Labeling Kit-SH, following the manufacturer’s protocols, and the labeled product (Ab15E9-biotin) was stored in a refrigerator. Fluorescent immunohistochemical staining (FICS) was performed using labeled indicators as described previously [34]. To detect S100A8 and S100A9 proteins in the rectal tissues, 3-µm-thick tissue sections were incubated with Ab2H6 (5 μg/mL) in a moisture chamber at 4 °C overnight, followed by incubation with TRITC-labeled anti-mouse IgG (5 μg/mL) for 1 h at room temperature. Subsequently, the sections were incubated with Ab15E9-biotin (5 μg/mL) at 4 °C overnight, followed by STA-FITC (5 μg/mL) for 1 h at room temperature. The stained tissues were mounted using the VECTASHIELD Antifade Mounting Medium containing DAPI, and images were obtained using a BIOREVO BZ-9000 microscope (Keyence Co., Ltd., Osaka, Japan).

### 2.9. ELISA

The S100A8 and S100A9 proteins in the rat colon tissues were measured using sandwich ELISA, in which two different antibodies were used to detect the target proteins, as previously described [34]. To detect S100A8 and S100A9, the primary antibodies, such as mAb2H6 (2 μg/mL) and mAb15E9 (2 μg/mL), respectively, were coated onto each well of the plate. HRP-conjugated mAb8A6 (2 μg/mL) and mAb10D11 (2 μg/mL) were used as secondary antibodies. An o-PD tablet was used as a substrate for color development. After termination with 3 M sulfuric acid, the absorbance at 490 nm was measured using an iMark™ microplate reader (Bio-Rad Laboratories, Inc.). The protein expression levels of S100A8 and S100A9 in the colon of each group were presented as relative amounts to those of the Normal group. Additionally, the levels of IL-6, IL-1β, and TNF-α in each sample were measured using ELISA kits, following the manufacturer’s protocols. The absorbance at 450 nm was measured using an iMark™ microplate reader (Bio-Rad Laboratories, Inc.). The cytokine concentrations in the large intestine were determined via conversion per gram of tissue.

### 2.10. PCR and Real-Time PCR

PCR and real-time PCR were performed to measure the cDNA in the colon tissue, as previously described [13]. PCR was performed using a C1000^TM^ thermal cycler (Bio-Rad Laboratories, Inc.). Agarose gel electrophoresis was performed using a Mupid-exU (Mupid Inc., Tokyo, Japan) to confirm amplification. Agarose gels were stained with ethidium bromide, and chemiluminescence was detected using a ChemiDoc XRS+ System (Bio-Rad Laboratories, Inc.). Real-time PCR was performed using the StepOnePlus Real-Time PCR System (Thermo Fisher Scientific) as previously described [13]. The mRNA expression levels in each sample were corrected and compared with those of β-actin concentration as the reference gene in the same sample. The results were presented relative to the expression level of the target gene in the Normal group (ΔΔCT method). The primers used in this study are listed in Table 1.

### 2.11. Assay Items of Clinical Chemistry in Rat Serum

Nineteen clinical chemistry assays in rat serum were submitted for analysis to the Nagahama Life Science Laboratory, Oriental Yeast Co., Ltd., Shiga, Japan. Equal volumes of serum samples from six rats per group were mixed to minimize individual differences. The assay was performed using JCA-BM6050 (JEOL Ltd., Tokyo, Japan) and included measurements of the total protein (TP), albumin (ALB), blood sugar (GLU), blood urea nitrogen (BUN), creatinine (CRE), uric acid (UA), iron (Fe), aspartate aminotransferase (AST), alanine aminotransferase (ALT), alkaline phosphatase (ALP), lactate dehydrogenase (LDH), amylase (AMY), creatine kinase (CK), γ-glutamyl transpeptidase (γ-GT), cholinesterase (ChE), lipase (Lip), total cholesterol (T-CHO), low-density lipoprotein cholesterol–cholesterol (LDL-C), and high-density lipoprotein cholesterol–cholesterol (HDL-C).

### 2.12. Isolation of Peritoneal MΦ from Rats

The peritoneal MΦ of the WT (*n* = 10) were isolated, as previously described [13]. The MΦ were elicited via the intraperitoneal injection of 10 mL of 4% thioglycolate in the DW. After 3 days, the elicited MΦ were collected in a 50 mL plastic tube using 50 mM sterilized PBS (buffer A). Subsequently, the tube was centrifuged (5 min, 1500 rpm, 4 °C), and the supernatant was discarded. The cells were suspended in 17 mM Tris-HCl (pH 7.2) containing 0.83% NH_4_Cl to hemolyze the contaminated erythrocytes before being incubated for 5 min at 37 °C. Following incubation, the cells were centrifuged, the supernatant was discarded, and residual cells were suspended in the Roswell Park Memorial Institute (RPMI)-1640 medium. A total of 2 × 10^6^ cells were plated in each well of a six-well plate prefilled with an appropriate volume of RPMI-1640 and incubated for 2 h in 5% CO_2_ at 37 °C. Following incubation, the non-adherent cells in each well were removed via washing thrice with buffer A, and the adherent cells were further incubated in the same medium in 5% CO_2_ at 37 °C.

### 2.13. Activation of MΦ and Confirmation of Their Immune Response

The MΦ were thoroughly washed with buffer A, resuspended in RPMI-1640 medium supplemented with S100A8, LPS, or anti-A8 antibody, and stimulated for 1 or 2 h. The assay conditions (1)–(6) were as follows:

(1) rr-S100A8 1 h: rr-S100A8 at 10 μg/mL was added to the medium and stimulated for 1 h;
(2) rr-S100A8 2 h: rr-S100A8 at 10 μg/mL was added to the medium and stimulated for 2 h;
(3) LPS 1 h: LPS at 10 μg/mL, was added to the medium and stimulated for 1 h;
(4) LPS 2 h: LPS at 10 μg/mL, was added to the medium and stimulated for 2 h;
(5) Anti-A8 1 h: an anti-A8 antibody at 10 μg/mL was added to the medium and incubated for 1 h;
(6) Anti-A8 → LPS 1 h: an anti-A8 antibody at 10 μg/mL was added to the medium and stimulated for 1 h. Subsequently, the medium was replaced with a medium containing 10 μg/mL of LPS and further stimulated for 1 h.

**Figure 2:**
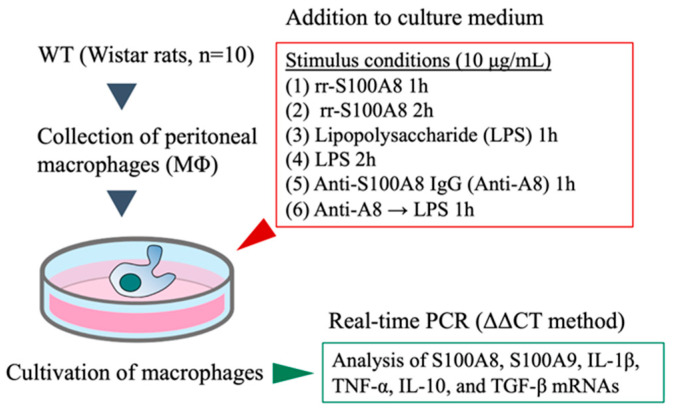
In vitro experimental protocol. Peritoneal MΦ of Wistar rats (*n* = 10) are isolated as previously described [13]. In vitro experiments were performed under six conditions (1)–(6). After stimulation, mRNAs of S100A8, S100A9, interleukin (IL)-1β, tumor necrosis factor-alpha (TNF-α), IL-10, and tumor growth factor (TGF)-β in macrophages (MΦ) were measured using real-time polymerase chain reaction (PCR) (ΔΔCT method).

The assay conditions (1)–(6) are schematically illustrated in Figure 2. After stimulation under each condition, MΦ were washed thrice with buffer A, and mRNA was subsequently extracted from the cells using TRIzol™ reagent. The mRNA expression levels of S100A8, S100A9, IL-1β, TNF-α, IL-10, and TGF-β in MΦ were measured using real-time PCR, which were corrected in comparison with that of β-actin in the cells. The results are expressed as the relative ratio to the gene expression level in MΦ without stimulation (ΔΔCT method).

### 2.14. Statistical Analysis

The significant difference between the groups was statistically identified using Mann–Whitney U, Kruskal–Wallis, and Tukey–Kramer tests. Furthermore, Dunnett’s test was also performed to identify statistical differences between the control group and the other ones. All the experiments were repeated five times, and the data were presented as the mean ± standard deviation (SD). The statistical significance was set at *p* < 0.05.

## 3. Results

Animal experiment ①

### 3.1. Alterations in Body Weight and Colon Length

**Figure 3:**
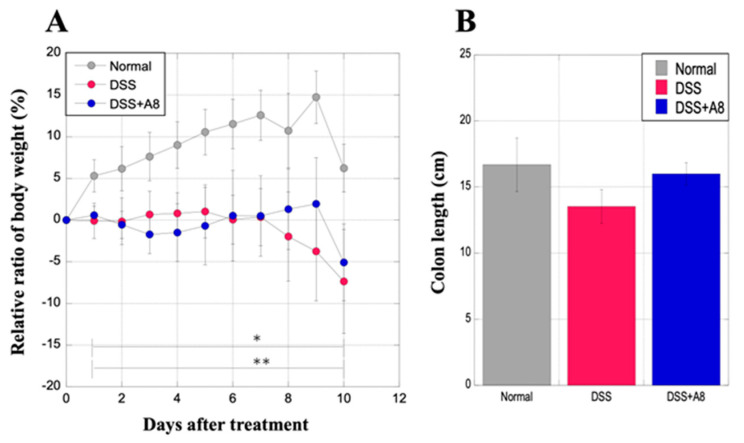
Alterations in the body weight and colon length of rats. (**A**) The *X*-axis represents the experimental period, whereas the *Y*-axis represents the percentage of fluctuation in the body weight of the three rat groups. (**B**) The *X*-axis represents three groups of rats, and the *Y*-axis represents the colon length (cm) of the three rat groups. Values are presented as the mean ± standard deviation (SD). * *p* < 0.05 (Normal vs. DSS), ** *p* < 0.05 (Normal vs. DSS + A8). Normal group (gray), DSS group (red), and DSS + A8 group (blue).

During this period, a gradual increase in body weight was observed in the rats of the Normal group, whereas the body weight in the DSS and DSS + A8 groups significantly decreased compared to that in the Normal group (Figure 3A). Additionally, the body weight of the rats in the DSS group appeared to be lower than that of the DSS + A8 group from days 8 to 10 after the initiation of the experiment (Figure 3A). However, these changes were not statistically significant. The colon length of the rats in the Normal and DSS + A8 groups were 16.68 ± 2.02 and 15.99 ± 0.85 cm (*p* = 0.712), respectively. The colon length of the rats in the DSS group was 13.53 ± 1.29 cm (*p* = 0.184), which was slightly shorter than that in the Normal group (Figure 3B).

### 3.2. Alterations in DAI Scores

**Figure 4:**
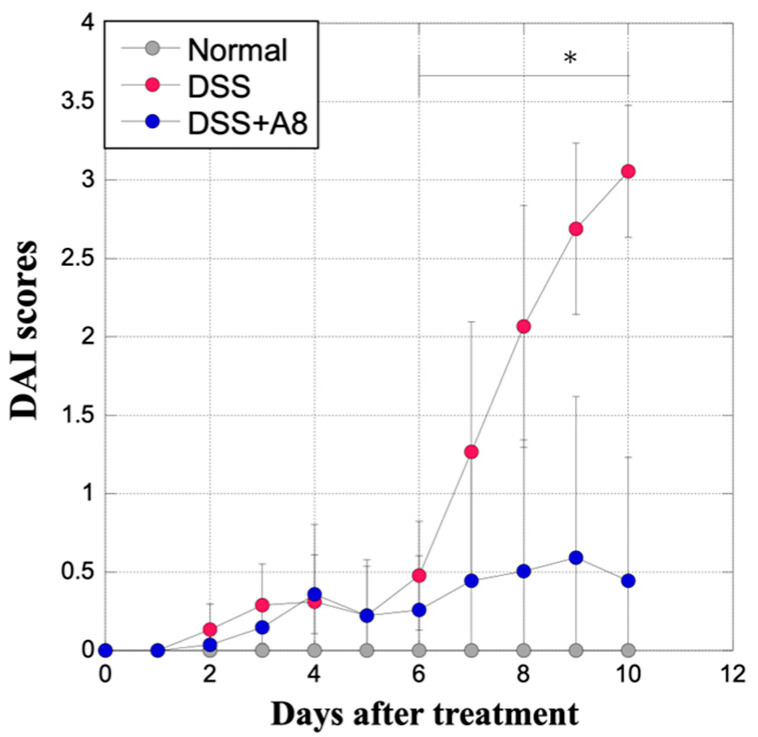
Alterations in the disease activity index (DAI) scores of rats. DAI scores in each group were assessed based on established criteria (Table 2) [31]. The *X*-axis represents the days after the start of the experiment, and the *Y*-axis represents the DAI scores [0–4]. Values are presented as the mean ± standard deviation (SD). * *p* < 0.05 (Normal vs. DSS). Normal group (gray); DSS group (red); and DSS + A8 group (blue).

The DAI score of the DSS group rapidly increased from days 6 to 10 at the start of the experiment, compared to that of the Normal group (Figure 4). In contrast, the DAI scores in the DSS + A8 group gradually increased during the experimental period, whereas no significant difference was observed between the Normal and the DSS + A8 groups (Figure 4). Specifically, the DAI scores in the DSS group from day 9 to 10 after the initiation of the experiment was higher than those in the DSS + A8 group (*p* = 0.031, 0.017) (Figure 4).

### 3.3. Microscopic Observation and Assessment of Colonic Inflammation in Animal Experiment ①

**Figure 5:**
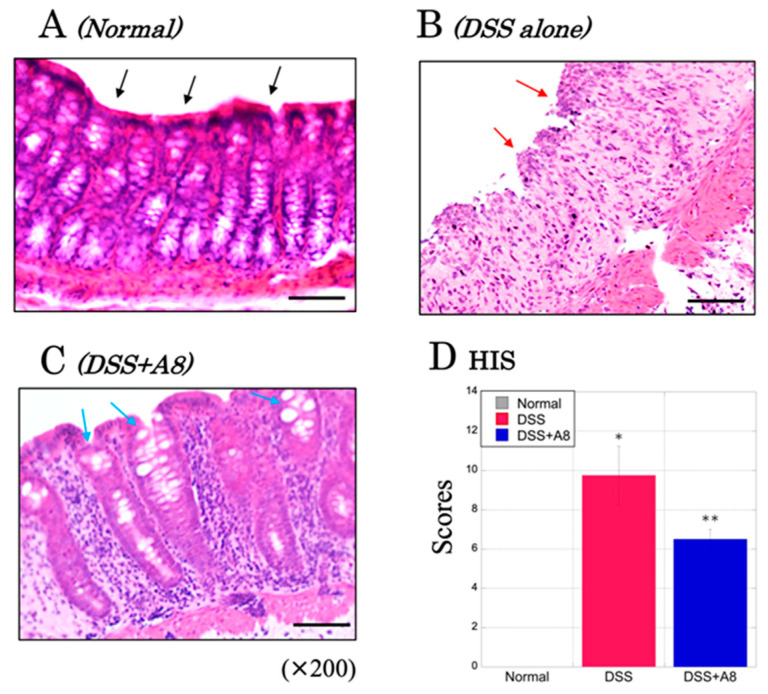
Histological severity (HIS) in the colonic tissues of rats. Panels (**A**–**C**) demonstrate microscopic images of the colon tissues in the Normal, DSS, and DSS + A8 groups, respectively, based on the hematoxylin and eosin (H&E) staining, in which all panels were observed with high power magnitude (×200). All microscopic images were observed using a BIOREVO BZ-9000 microscope (Keyence Co., Ltd., Osaka, Japan). Black arrows; non-inflammatory regions, red arrows; inflammatory regions, and light blue arrows; mild inflammatory regions. Black scale bar = 50 μm. Panel (**D**) demonstrates HIS scores, which are assessed based on established criteria (Table 3) on day 10 after initiating the experiment [32]. The *y*-axis represents the HIS scores [0–14]. Values are presented as the mean ± standard deviation (SD). * *p* < 0.05 (Normal vs. DSS), ** *p* < 0.05 (Normal vs. DSS + A8). Normal group (gray); DSS group (red); and DSS + A8 group (blue).

In the colons of the rats in the Normal group, the mucosal epithelium was normal with no damage, and a large number of goblet cells were observed (Figure 5A, black arrows). In contrast, in the DSS group, the mucosal epithelium was distinctly damaged and immune cells infiltrated around the goblet cells (Figure 5B, red arrows). The DSS + A8 group demonstrated a minimal loss of epithelial and goblet cells and less immune cell infiltration than that of the DSS group (Figure 5C, light blue arrows). The HIS scores of the DSS group (9.75 ± 1.48, *p* = 0.001) and the DSS + A8 group (6.50 ± 0.51, *p* = 0.002) were significantly higher than those of the Normal group (Figure 5D). In addition, the comparisons between the DSS and DSS + A8 groups showed that the former had significantly higher HIS scores (*p* = 0.024).

### 3.4. Assessment of S100A8 and S100A9 Expression in Rat Colon

**Figure 6:**
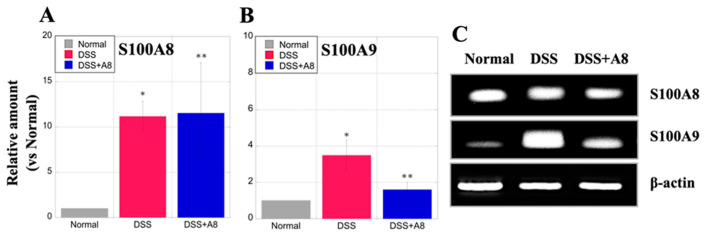
Analysis of S100A8 and S100A9 expressions in colon tissue using enzyme-linked immunosorbent assay (ELISA). Panels (**A**,**B**) demonstrate the relative amount of S100A8 and S100A9, respectively. The *Y*-axis represents the relative amount of the expression level (absorbance) of S100A8 and S100A9 in the colon tissue of the Normal group as “1”. Values are presented as the mean ± standard deviation (SD). * *p* < 0.05 (Normal vs. DSS), ** *p* < 0.05 (Normal vs. DSS + A8). DSS group (red) and DSS + A8 group (blue). Panel (**C**) demonstrates the mRNA expression levels of S100A8 (upper panel) and S100A9 (middle panel) in the colon. The expression level of β-actin (lower panel) in the colon is used as an internal control. Images are captured using a ChemiDoc XRS+ (Bio-Rad Laboratories, Inc.). The images of the original PCR and agarose gel electrophoresis results are shown in Figure S1.

The relative amount of S100A8 in the DSS (11.19 ± 1.66, *p* = 0.001) and DSS + A8 groups (11.54 ± 5.59, *p* = 0.001) was >10-fold higher than that in the colon tissue of the Normal group (Figure 6A). Furthermore, the relative amount of S100A9 in the colon of rats in the DSS (3.49 ± 0.83, *p* = 0.011) and DSS + A8 groups (1.61 ± 0.39, *p* = 0.041) were slightly higher than that in the Normal group (Figure 6B). Furthermore, comparisons between the DSS and DSS + A8 groups showed that the former had significantly higher S100A9 expression (*p* = 0.035) (Figure 6B). The agarose gel electrophoresis assay demonstrated a minimal difference in the mRNA expression of S100A8 in colon tissue among the three groups (Figure 6C, upper panel). In contrast, the S100A9 mRNA expression in the colon tissue was significantly upregulated in the DSS group, whereas it was slightly upregulated in the DSS + A8 group (Figure 6C, middle panel).

### 3.5. Analysis of S100A8 and S100A9 Expression in Colon Tissue Using FICS

**Figure 7:**
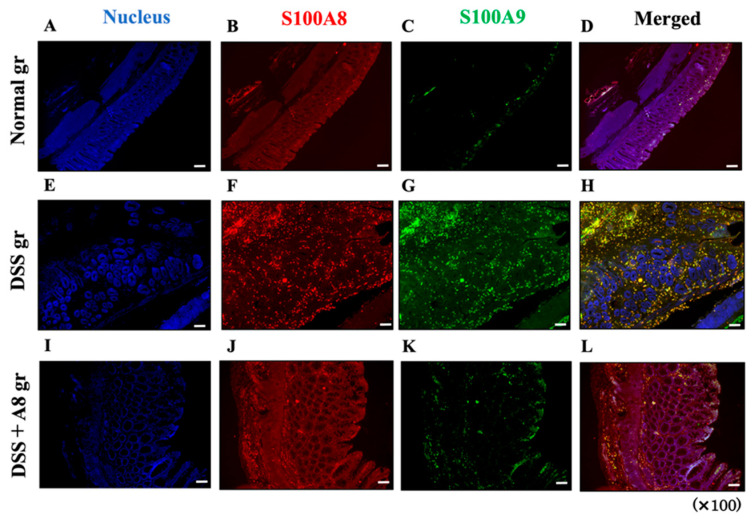
Fluorescent immunohistochemical staining (FICS) of S100A8 and S100A9 in rat colon tissues. Expression of S100A8 (red, tetramethylrhodamine [TRITC]) and S100A9 (green, FITC) in colon tissues were assessed, in which nuclei (blue, 4′,6-diamidino-2-phenylindole [DAPI]) are counterstained. Panels (**A**–**D**) (Normal); panels (**E**–**H**) (DSS); and panels (**I**–**L**) (DSS + A8). Panels (**A**,**E**,**I**) (DAPI); panels (**B**,**F**,**J**) (TRITC); panels (**C**,**G**,**K**) (FITC); and panels (**D**,**H**,**L**) (merged). All microscopic images were obtained using a BIOREVO BZ-9000 microscope (Keyence Co., Ltd., Osaka, Japan) with high power magnitude (×100). White scale bar = 50 μm.

In the Normal group, the colon tissues of the rats demonstrated poor S100A8 and S100A9 expression (Figure 7, panels A–D). However, they were highly expressed in the colon tissue of the DSS group, with nearly overlapping localizations (Figure 7, panels E–H). Additionally, the expression levels of S100A8 and S100A9 in the colon tissues of the DSS + A8 group were lower than those in the DSS group (Figure 7, panels I–L).

### 3.6. Analysis of NE Expression in Colon Tissue

**Figure 8:**
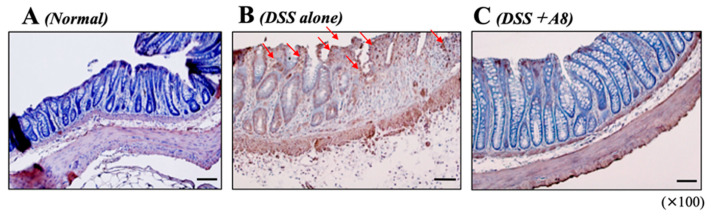
Immunohistochemical (IHC) staining of neutrophil elastase (NE) in colon tissues. The localization of neutrophils in the colon tissues of Normal (**A**), DSS (**B**), and DSS + A8 (**C**) groups was observed by detecting NE expression in neutrophils. Positive reactions (brown, red arrows) are stained with diaminobenzidine (DAB). All microscopic images are observed using a BIOREVO BZ-9000 microscope (Keyence Co., Ltd., Osaka, Japan) with high power magnitude (×100). Black scale bar = 50 μm.

In the colon tissues of the Normal (Figure 8A) and DSS + A8 groups (Figure 8C), a few positive reactions to NE were observed. In contrast, a strong positive reaction to neutrophil elastase (NE) was observed in the colon tissues of the DSS group, specifically in the mucosal epithelium (Figure 8B, red arrows).

### 3.7. Measurement of Inflammatory Cytokines in the Serum and Colon Tissue of Rats Using ELISA

**Figure 9:**
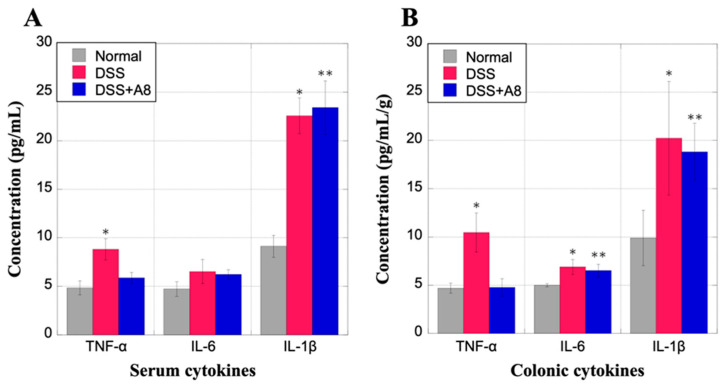
Measurement of inflammatory cytokines in the serum and colon tissue of rats using enzyme-linked immunosorbent assay (ELISA). Inflammatory cytokines (tumor necrosis factor [TNF]-α, interleukin [IL]-6, and IL-1β) in the serum (panel (**A**)) and colon tissue (panel (**B**)) were measured using ELISA. The *Y*-axis represents the concentration of each cytokine (pg/mL or pg/mL/g). Values are presented as the mean ± standard deviation (SD). * *p* < 0.05 (Normal vs. DSS), ** *p* < 0.05 (Normal vs. DSS + A8). Normal group (gray); DSS group (red); and DSS + A8 group (blue).

The concentration of TNF-α in the serum samples was significantly higher in the DSS group (8.81 ± 1.07 pg/mL, *p* = 0.039) than that in the Normal (4.91 ± 1.41 pg/mL) and the DSS + A8 group (5.87 ± 0.56 pg/mL, *p* = 0.106) (Figure 9A). In contrast, the IL-6 in the serum samples exhibited no significant differences in their concentration between the DSS (6.53 ± 1.25, *p* = 0.057), DSS + A8 (6.22 ± 0.50, *p* = 0.051), and the Normal groups (4.86 ± 1.38 pg/mL) (Figure 9A). The concentration of the IL-1β in the serum samples was significantly higher in the DSS (22.57 ± 1.85 pg/mL, *p* = 0.004) and DSS + A8 groups (23.42 ± 2.74 pg/mL, *p* = 0.004) than that in the Normal group (8.22 ± 2.01 pg/mL) (Figure 9A). Additionally, the TNF-α levels in the colonic samples were higher in the DSS group (10.45 ± 2.04 pg/mL/g, *p* = 0.015) than those in the Normal (4.65 ± 0.51 pg/mL/g) and DSS + A8 group (4.76 ± 0.90 pg/mL/g, *p* = 0.912) (Figure 9B). The IL-6 concentrations in the colonic samples were slightly higher in the DSS (6.89 ± 0.77 pg/mL/g, *p* = 0.026) and DSS + A8 (6.51 ± 0.66 pg/mL/g, *p* = 0.031) groups, compared to the Normal group (5.02 ± 0.09 pg/mL/g) (Figure 9B). Similarly, the IL-1β levels in the colonic samples were significantly higher in the DSS (20.22 ± 5.88 pg/mL/g, *p* = 0.039) and DSS + A8 (18.8 ± 2.96 pg/mL/g, *p* = 0.019) groups, compared to the Normal group (9.92 ± 3.51 pg/mL/g) (Figure 9B). As described above, overall, the expression levels of TNF-α, IL6, and IL1β showed similar trends in the serum and colon samples from each group.

### 3.8. Assay Items of Clinical Chemistry in Rat Serum in Three Groups

The concentrations of TP, ALB, T-CHO, and HDL-C in the DSS group were lower than those in the Normal group (Table 4). In contrast, the AST and CK activities in the DSS group were higher than those in the Normal group (Table 4). Unfortunately, these parameters were not normalized in the DSS + A8 group either (Table 4). Furthermore, the concentration of Fe in the serum was markedly reduced in the DSS + A8 group, compared to that in the DSS group (Table 4).

Animal experiment ②

### 3.9. Alterations in the Body Weight and Colon Length of Rats in Each Group

**Figure 10:**
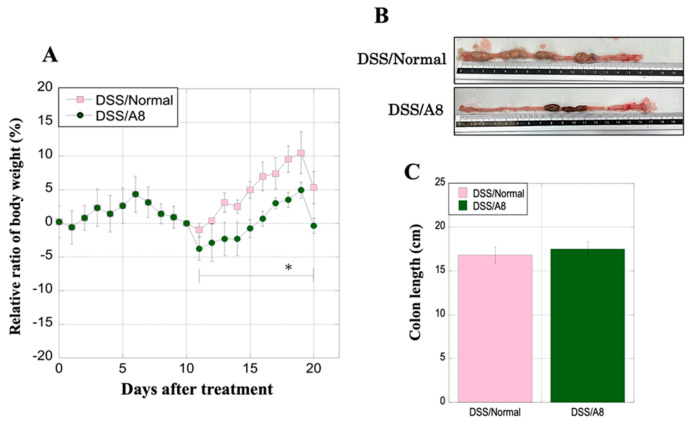
Alterations in the body weight and colon length of rats. (**A**) The *X*-axis represents the experimental period, and the *Y*-axis represents the percentage of alteration in the body weight of rats in the two groups. (**B**) Pictures of representative colon tissues from the DSS/Normal (upper panel) and DSS/A8 (lower panel) groups. (**C**) The *Y*-axis represents the colon length (cm) of the rats in the two groups. Values are presented as the mean ± standard deviation (SD). DSS/Normal group (pink) and DSS/A8 group (green).

In a protocol comparing two groups that switched to drinking DW for 10 days after a 10-day period of drinking 3% DSS, the DSS/S100A8 group exhibited a slight loss in body weight than that of the DSS/Normal group (Figure 10A). However, there was a minimal difference in the colon length between the two groups (16.8 ± 0.92 and 17.5 ± 0.83 cm, respectively, *p* = 0.895) (Figure 10B,C).

### 3.10. Alterations in DAI Scores of Rats

**Figure 11:**
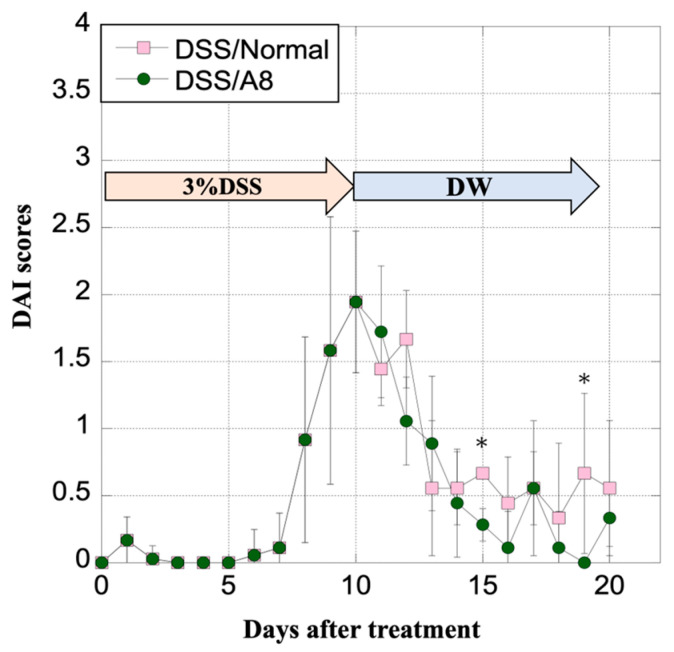
Alterations in the disease activity index (DAI) scores of rats. DAI scores were assessed based on established criteria (Table 2) [31]. The *Y*-axis represents the DAI scores [0–4] on every experimental day. Values are presented as the mean ± standard deviation (SD). * *p* < 0.05 (DSS/Normal vs. DSS/A8). DSS/Normal group (pink) and DSS/A8 group (green).

In both the DSS/Normal and DSS/A8 groups, the DAI score was at its maximum on day 10 after the administration of 3% DSS, and then gradually reduced (Figure 11). In the latter half of the experimental period, the DAI scores were significantly reduced in the DSS/Normal and DSS/A8 groups; however, the scores in the latter group were lower than those in the former group (Figure 11).

### 3.11. Microscopic Observation and Assessment of Colonic Inflammation in Animal Experiment ②

**Figure 12:**
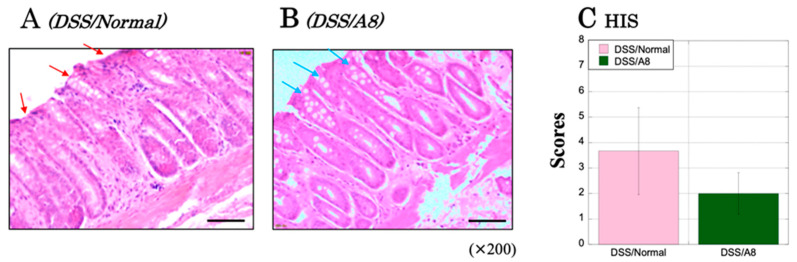
Histological assessment of the severity in colonic tissues of rats based on hematoxylin and eosin (H&E) staining. Panels (**A**,**B**) demonstrate microscopic images of the colon tissues in DSS/Normal and DSS/A8 groups, respectively. Red arrows; inflammatory regions and light blue arrows; mild inflammatory regions. All the microscopic images are observed using a BIOREVO BZ-9000 microscope (Keyence Co., Ltd., Osaka, Japan). All the panels are observed at high magnification (×200). Black scale bar = 50 μm. Panel (**C**) demonstrates histological severity (HIS) scores, which are assessed based on the established criteria, on day 20 after initiating the experiment [32]. The *Y*-axis represents the HIS scores [0–14]. Values are presented as the mean ± standard deviation (SD). DSS/Normal group (pink) and DSS/A8 group (green).

In the colon of the DSS/Normal group, the slight loss of mucosal epithelium, the loss of goblet cells, and the extensive infiltration of immune cells were observed (Figure 12A, red arrows). In contrast, certain inflammatory alterations were observed in the DSS/A8 group; however, overall, they were scarce (Figure 12B, light blue arrows). Based on the results of H&E staining, the HIS scores based on the criteria are summarized in Table 3 [33]. More HIS scores were restrained in the DSS/A8 group (2.00 ± 0.82) than in the DSS/Normal group (3.67 ± 1.70, *p* = 0.349) (Figure 12C).

### 3.12. S100A8 and S100A9 mRNA Expression in MΦ

**Figure 13:**
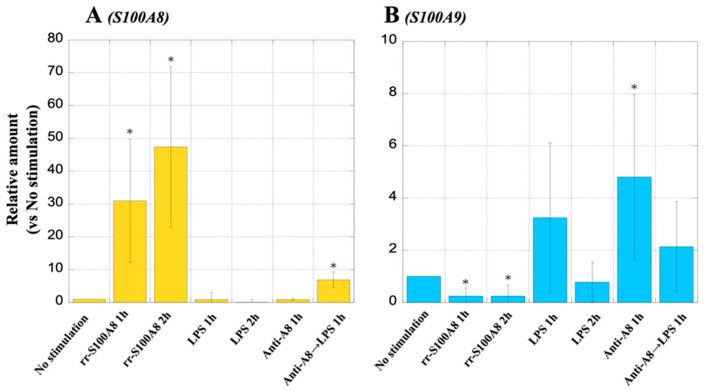
Analysis of S100A8 and S100A9 mRNA expression in macrophages (MΦ) using real-time polymerase chain reaction (PCR). The mRNA expression levels of the S100A8 and S100A9 are indicated by panels (**A**,**B**), respectively. *Y*-axis represents the relative amount of the expression level of S100A8 and S100A9 mRNAs in the MΦ as the expression in the cells with no stimulation as “1”. Values are presented as the mean ± standard deviation (SD). * *p* < 0.05 (No stimulation vs. each stimulus condition).

After MΦ were stimulated with rr-S100A8 for 2 h, the S100A8 mRNA expression in the MΦ was significantly induced (47.41 ± 24.44, *p* = 0.001), compared to no stimulation MΦ. However, upon the stimulation of the MΦ with LPS for 2 h, the S100A8 mRNA expression (0.07 ± 0.10, *p* = 0.911) was rather reduced compared to unstimulated MΦ. (Figure 13A). In addition, when the MΦ were stimulated with rr-S100A8 for 2 h, the S100A9 mRNA expression (0.24 ± 0.43, *p* = 0.035) was more suppressed than in the unstimulated MΦ (Figure 13B). When the MΦ were stimulated with LPS for 1 h, the S100A9 mRNA expression (3.24 ± 2.86, *p* = 0.22) tended to increase compared to no stimulation; however, it reduced over time (Figure 13B). The S100A9 mRNA expression in MΦ was strongly induced through anti-A8 treatment (4.80 ± 3.17, *p* = 0.043) (Figure 13B).

### 3.13. Analysis of Cytokine mRNA Expression in MΦ

**Figure 14:**
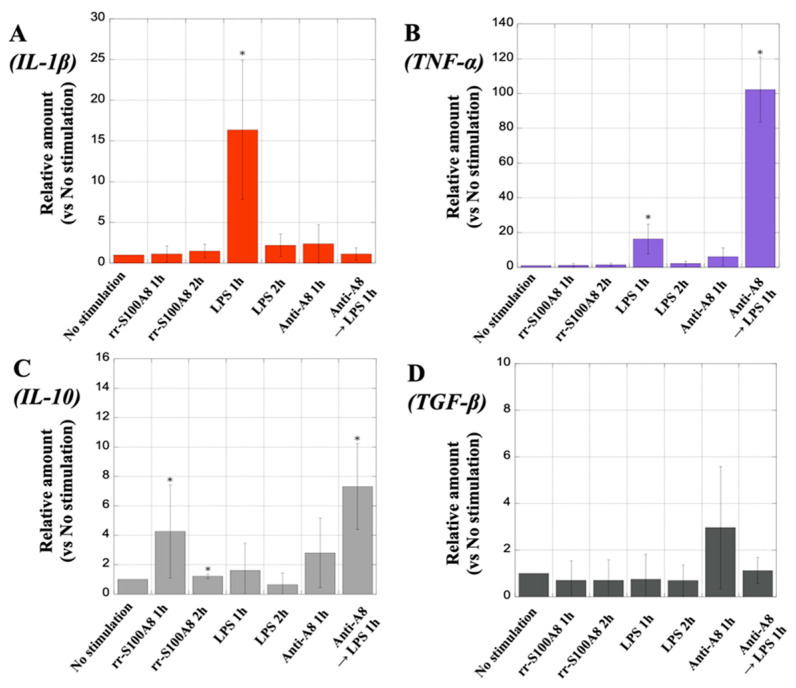
Analysis of cytokine mRNA expression in macrophages (MΦ) using real-time polymerase chain reaction (PCR). Panels (**A**–**D**) demonstrate interleukin (IL)-1β, tumor necrosis factor (TNF)-α, IL-10, and tumor growth factor (TGF)-β mRNA levels, respectively. The *Y*-axis represents the relative amount against the expression level of each cytokine in MΦ with no stimulation as referred to as “1”. Values are presented as the mean ± standard deviation (SD). * *p* < 0.05 (no stimulation vs. each stimulus condition).

When the MΦ were stimulated with rr-S100A8 for 2 h, the IL-1β expression in the MΦ (1.48 ± 0.85, *p* = 0.398) was comparable to that of the unstimulated MΦ (Figure 14A). In contrast, upon the stimulation of the MΦ with LPS for 1 h, the IL-1β levels in the MΦ were significantly increased (16.4 ± 8.56, *p* = 0.006); however, anti-A8 did not affect the MΦ. Although TNF-α expression in the MΦ was not induced by stimulation with rr-S100A8 for 2 h (1.24 ± 0.81, *p* = 0.695) compared to no stimulation, it was significantly induced by LPS stimulation for 1 h (16.9 ± 8.56, *p* = 0.005) (Figure 14B). Additionally, the TNF-α levels in the MΦ significantly increased upon the stimulation of the MΦ with LPS following anti-A8 treatment (102.26 ± 18.60, *p* = 0.001), compared to the unstimulated MΦ. The stimulation of the MΦ with rr-S100A8 for 1 h significantly induced IL-10 expression (4.21 ± 3.56, *p* = 0.048) compared to no stimulation (Figure 14C). In contrast, after stimulating the MΦ with LPS for 1 h, the IL-10 levels in the MΦ hardly increased (1.80 ± 1.86, *p* = 0.727). Additionally, the IL-10 levels were increased by treating the MΦ with an anti-A8 antibody (2.89 ± 2.58, *p* = 0.011), compared to the unstimulated MΦ. The mRNA expression of the TGF-β in the MΦ did not show significant fluctuations under any stimulation conditions. (Figure 14D).

## 4. Discussion

In this study, we aimed to assess the pharmacological effects of S100A8 in treating UC by intraperitoneally administering rr-S100A8 to rats with DSS-induced experimental colitis. It has been widely reported that S100A8/A9, the heterodimer of S100A8 and S100A9, has a causal relationship with UC, and that fluctuations in its concentration in stool are a useful index of UC severity [19]. S100A9 activates specific signaling pathways and is primarily involved in UC exacerbation [22,23]. However, the functional role of S100A8 in UC remains unclear. Our attempts to elucidate the immune function of S100A8 are crucial in the field of immunology. Therefore, we hypothesized that S100A8 functions as an anti-inflammatory protein and is involved in the regulation of UC in an intricate manner.

The DAI score results indicate that rr-S100A8 may serve as an immunosuppressive drug that regulates excessive immune responses in the colon tissues of rats with experimental colitis. Although the mechanism of negative regulation by rr-S100A8 remains unclear, S100A8 may inhibit the inflammatory signal transduction pathway in rats with exacerbated UC. Microscopic images of colon tissue from the rr-S100A8-treated experimental UC model rats demonstrated only a slight loss of the mucosal epithelium. This indicates that rr-S100A8 suppresses the progression of experimental colitis. Although immune cell infiltration was still observed in the colon tissues of experimental UC rats, it was minimal despite treatment with rr-S100A8. However, no significant immune responses were observed in the DSS + A8 group. This strongly indicates that rr-S100A8 reduces excessive activation of immune cells in the colon tissue and can serve as an immunosuppressive agent in rats with experimental colitis.

The majority of intraperitoneally administered substances generally permeate peritoneal mesothelial cells, which are absorbed by capillaries in connective tissue and intercellular spaces, and enter the systemic circulatory system through the portal veins [35]. Additionally, it has been speculated that rr-S100A8 migrates into the systemic circulatory system to exert its role(s) as an immunological suppressor. Therefore, rr-S100A8 may suppress the excessive activation of neutrophils, monocytes, MΦ, and lymphocytes in the blood, thereby alleviating experimental colitis. Alternatively, STAT3 activation and increased S100A9 levels in the colonic tissues of exacerbated UC have been extensively reported [22]. Considering the PCR and real-time PCR results in colorectal tissues, it may be speculated that S100A8 reduces the excessive expression of S100A9 by negatively regulating the exacerbation of inflammation. The localization of S100A8 in the colon tissue of rats with UC closely resembled that of S100A9, indicating that S100A8 may bind to S100A9, which is overexpressed in inflammatory tissues, to prevent disease progression. Therefore, S100A8 and S100A9 may regulate immune function by reversibly binding and maintaining intestinal homeostasis. The heterodimer S100A8/A9 may be excreted in the stool as a result of the immune reaction involving S100A8 and S100A9 in the intestinal tract. These hypotheses are consistent with previous papers reporting S100A8/A9 as a biomarker for UC [19], and our experiments are significant in that we found similar phenomena in basic research. In particular, we have an advantage in that we have rr-S100A8 and its antibodies, and there are few similar examples of this study in the world, which may have led to these useful results.

NE, an enzyme produced in activated neutrophils, is anticipated to be a specific biomarker for UC because of the observed infiltration of numerous NE-positive neutrophils in the colonic tissue of patients with UC [36,37]. Because S100A8 and S100A9 are expressed in neutrophils, it is speculated that numerous neutrophils that infiltrate inflammatory tissues regulate the production and secretion of these two proteins [1,2]. The administration of S100A8 may contribute to a significant reduction in TNF-α levels in both serum and colon tissues. TNF-α, a potent inflammatory mediator, activates various transcription factors, such as c-Jun N-terminal kinase and nuclear factor-kappa B, in immune cells [38]. These transcriptional responses facilitate the expression of adhesion factors, such as intercellular cell adhesion molecule-1 and vascular cell adhesion molecule-1 that induce the local infiltration of inflammatory cells, such as neutrophils and MΦ, from circulating blood [38]. Based on these results, S100A8 may suppress TNF-α transcription and production, thereby indirectly preventing excessive immune cell infiltration in the colon tissues. Experimental data obtained using rat sera indicated that the intraperitoneal administration of rr-S100A8 had both positive and negative immunosuppressive effects. This is the first attempt in the world to examine the effects of rr-S100A8 administration on the whole body of rats by analyzing serum biochemical laboratory data. The AST and CK levels are representative indicators of liver and muscle damage, respectively. The reduction in the serum AST and CK levels in the rats following rr-S100A8 administration indicated that worsening UC may strain the liver and muscles, resulting in functional decline, whereas S100A8 may alleviate them. However, a reduction in the concentration of serum TP, ALB, Fe, and T-CHO following rr-S100A8 administration indicates that S100A8 may be involved in the malabsorption and reduced synthesis of dietary nutrients. For future clinical applications of rr-S100A8 as a therapeutic agent for UC, further assessment is required to clarify its long-term effectiveness or adverse effects.

In animal experiment ①, rats were free to drink 3% DSS and simultaneously received an intraperitoneal administration of rr-S100A8 during the experimental period. This approach enabled us to observe the effects of rr-S100A8 on acute colitis in rats. UC is a recurrent disease that requires effective treatment strategies for remission. We assessed whether the intraperitoneal administration of rr-S100A8 has a therapeutic effect and whether it accelerates the remission of experimental UC despite its development in rats. The body weights remained low even after rr-S100A8 administration, indicating that rr-S100A8 is not always effective in treating experimental colitis because of toxicity or poor nutrient absorption. Additionally, regardless of the presence or absence of rr-S100A8 administration, no significant alterations were observed in the colon length, indicating that rr-S100A8 did not participate in the remission of experimental colitis. Therefore, S100A8 does not directly repair experimental colitis in rats but may contribute to the negative regulation of the excessive activation of immune cells associated with acute inflammation. Additionally, the DAI score demonstrated that the intraperitoneal administration of rr-S100A8 was ineffective in repairing remission in experimental UC. This is because treatment with rr-S100A8 did not significantly suppress diarrhea and hemorrhage in the experimental UC rats in comparison to the spontaneous remission of colitis when 3% DSS was replaced with DW in the experiment. In animal experiment ②, the peritoneal administration of rr-S100A8 after the onset of UC resulted in a slight histopathological enhancement in colitis. Therefore, the peritoneal administration of rr-S100A8 may be partially effective for remission in experimental colitis, although the reason for its limited remission remains unclear.

We discussed the results of the in vivo experiments and indicated that rr-S100A8 may moderately suppress acute intestinal inflammation. In this study, rr-S100A8 partially resembled the immunosuppressive agents. However, it is challenging to confirm the involvement of S100A8 in the immune cells of UC rats through in vivo experiments. We conducted in vitro experiments using rat abdominal cavity-derived MΦ to confirm the S100A8-induced autocrine signal transduction pathway. In this pathway, cells or allogeneic cells that release active substances were repeatedly activated under their influence. In this study, we confirmed that the activity of intracellular S100A8 was significantly enhanced by adding rr-S100A8 to the MΦ culture medium. We previously demonstrated that S100A8 and S100A9 have an autocrine activation pathway within MΦ [7,34]. Although the LPS-stimulated MΦ did not induce S100A8 expression, they exhibited significant S100A9 expression. LPS is primarily observed in the outer membrane of Gram-negative bacteria and activates MΦ in a proinflammatory manner through receptors, such as the cluster of differentiation (CD)14, CD18, and TLR-4 [39]. Additionally, it is expressed in certain bacteria belonging to the Enterobacteriaceae group that coexist in large colon tissues, and its relevance to UC pathogenesis has been of significant concern. The activation of MΦ by LPS reduces S100A8 and increases S100A9, indicating the likely role of S100A9 in signal transduction, resulting in inflammatory deterioration. Additionally, S100A9 is significantly more expressed than that of S100A8 in anti-A8-treated MΦ. Our previous findings demonstrated that the treatment of MΦ with anti-A8 inhibits S100A8 expression [34]. Therefore, the suppression of S100A8 expression in MΦ possibly increases S100A9 expression, indicating that S100A8 may serve as an antagonist for S100A9, indirectly contributing to the alleviation of inflammation.

In contrast, after treating the MΦ with anti-A8, the intracellular S100A8 and S100A9 levels did not significantly increase, despite subsequent activation by LPS. Although the reason for this remains unclear, we anticipate that the compensatory mechanism of the organism counteracted LPS-induced MΦ activation because the S100A8 in the MΦ was temporarily inhibited by anti-A8. The excessive activation of S100A8 or S100A9 may result in immunological abnormalities [34]. Therefore, even if S100A9 expression is temporarily increased by anti-A8, the delayed and compensatory activation of S100A8 is predictive, because S100A8 maintains the immunological homeostasis of the organism. Subsequently, we assessed the effects of the rr-S100A8 and LPS stimulation of MΦ on the production of inflammatory cytokines. These results indicate that the stimulation of MΦ by rr-S100A8 did not enhance the production of inflammatory cytokines, such as IL-1β and TNF-α. Additionally, they indicated that S100A8 may not act in an inflammation-aggravating manner. Moreover, LPS stimulation significantly increased TNF-α expression in the anti-A8-treated MΦ. Animal experiment ① demonstrated that rr-S100A8 reduced the TNF-α levels during inflammation. Therefore, S100A8 likely inhibits the production and secretion of inflammatory cytokines, specifically TNF-α. Although the reason for the S100A8-induced suppression of TNF-α expression remains unclear, the binding of LPS to various receptors may alternatively facilitate the expression of S100A9 and its activation through the proinflammatory signaling pathway.

Based on these results, S100A8 may play a crucial role(s) in suppressing inflammation by inhibiting the aforementioned pathway. This hypothesis indicates that S100A8 binds to the S100A9 inside MΦ to form the S100A8/A9 heterodimer, which blocks the proinflammatory signaling pathway and is subsequently excreted out of the body. Additionally, we assessed the effect of rr-S100A8-induced MΦ stimulation on the production of anti-inflammatory cytokines. rr-S100A8 facilitated IL-10 expression in the MΦ. Additionally, the treatment of the MΦ with an anti-A8 antibody and LPS induced IL-10 expression in a similar manner. As estimated, it appeared that IL-10 was produced to control the exacerbation of inflammation via the LPS stimulation of MΦ. Moreover, IL-10 was reactively released from MΦ in response to stimuli by rr-S100A8, the anti-A8 antibody, and LPS. Therefore, the significance of IL-10 activity in MΦ may be limited. Similarly, the TGF-β activity did not significantly differ under each stimulus condition, and all were less active. Therefore, no specific association was observed between the TGF-β and S100A8 in the MΦ. Based on this discussion, it appears unlikely that S100A8 contributes to the increased production and secretion of IL-10 and TGF-β, or directly ameliorates UC. Because of the ongoing global controversies regarding the factors affecting UC onset [40], further assessment is required.

Increased S100A8 levels in the intestinal tract accelerate helper T cell hyperactivity and exacerbate DSS-induced colitis [25]. An anti-A8 antibody was successfully administered to rats to maintain S100A8 expression in the intestinal tract at low levels and prevent severe colitis [25]. Although this may contradict our results, excessive S100A8 expression may be detrimental to the pathogenesis of inflammatory diseases other than UC. Therefore, S100A8 may be a cytokine-like substance, and its immunoregulatory mechanisms may be regulated based on its concentration. However, despite the induction of colitis by DSS, its pathogenesis in transgenic animals (Tg-S100A8) was alleviated [24]. Additionally, the TNF-α levels were reduced in the rectal region of the DSS-treated Tg-S100A8. These results indicate a negative correlation between S100A8 and TNF-α [24]. This study strongly supports the validity of our results. An advantage of this study was the use of a large amount of rr-S100A8 produced in *E. coli* cells through genetic technology. Basic research using Tg-S100A8 is crucial for assessing the essential role of S100A8 in the immune system; however, its clinical application in patients with UC is currently premature. In contrast, this study offers an excellent protocol for animal experiments that can be translated into actual practice, such as determining whether the external administration of rr-S100A8 inhibits UC exacerbations or contributes to its alleviation. If further studies confirm the anti-inflammatory effects of S100A8 in treating inflammatory diseases, its clinical applications in treating UC will be confirmed.

In this study, we treated rats with UC, and the results obtained were useful for clarifying the pharmacological effects of S100A8. The strength of our research is the large supply of rr-S100A8, which could be exploited to pursue the essential role of the S100A8 protein. However, this study has certain limitations. First, it is unclear whether the function of rr-S100A8 in rats is similar to that of the S100A8 protein in humans. To investigate this, it is necessary to supply human recombinant S100A8 and scrutinize its immunological role. Second, the dose of rr-S100A8 administered to the rats was fixed at 1.0 mg/kg only. This indicated that the peptide concentration, which provided a favorable pharmacological effect on the UC model rats, was determined in the previous studies and was >0.6 mg/kg [41]. However, the dose effect of rr-S100A8 should be assessed at concentrations <1.0 mg/kg because rr-S100A8 may exert its immune function in a concentration-dependent manner. Additionally, there is potential for optimizing the concentration of the DSS administered. During the study period, we observed slight weight loss, diarrhea, and blood loss, which were mostly non-severe in rats. In this study, the concentration of DSS used to observe significant differences in the presence or absence of rr-S100A8 may have been inadequate. Therefore, we believe that increasing the concentration of DSS and rr-S100A8 administered or extending the administration period may have resulted in a clear difference between the rat groups. Additionally, other concentrations should be assessed with the stimulating concentrations (10 μg/mL) of rr-S100A8, LPS, and anti-A8 added to the medium in the in vitro experiments. Similar results at different concentrations may offer robust evidence for the functional role of S100A8. To elucidate the essential functional role of S100A8 in detail, further assessment is required to address these challenges.

## 5. Conclusions

S100A8 suppresses the intracellular signaling pathway of exacerbated inflammation involving LPS and S100A9 during UC inflammation. By blocking this pathway, S100A8 serves as an anti-inflammatory protein that specifically regulates the production and secretion of the proinflammatory cytokine TNF-α. Additionally, we hypothesized that S100A8 plays a functional role in blocking signal transduction by binding to S100A9 and that S100A8/A9 is the product of this reaction. This study enabled us to speculate the immunological role of S100A8.

## Figures and Tables

**Table 1:** List of the primers used in this study.

	Gene	Forward	Reverse	Size (bp)
For PCR	β-Actin	5′-CTACAATGAGCTGCGTGTGG-3′	5′-CTCCGGAGTCCATCACAATG-3′	200
	S100A8	5′-GCAACTGAACTGGAGAAGGC-3′	5′-GACATATCCAAGGGCCCAG-3′	308
	S100A9	5′-GACATCCTGACACCCTGAACAAG-3′	5′-CCCATCAGCATCATACACTCCTC-3′	171
For Real-Time PCR	β-Actin	5′-TGTGTTGTCCCTGTATGCCTCTG-3′	5′-ATAGATGGGCACAGTGTGGGTG-3′	85
	S100A8	5′-ACTGGAGAAGGCCTTGAGCAAC-3′	5′-ATCCCTGTAGAGGGCATGGTGA-3′	85
	S100A9	5′-TCATGGAGGACCTGGACACAAA-3′	5′-GCAGCTTCTCATGACAGGCAAA-3′	99
	IL-1β	5′-CACCTCTCAAGGAGAGCACAGA-3′	5′-ACGGGTTCCATGGTGAAGTC-3′	81
	IL-10	5′-CCCTCTGGATACAGCTGCG-3′	5′-GCTCCACTGCCTTGCTTTTATT-3′	69
	TNF-α	5′-GTGATCGGTCCCAACAAGGA-3′	5′-AGGGTCTGGGCCATGGAA-3′	71
	TGF-β	5′-ACCTGCAAGACCATCGACATG-3′	5′-CGAGCCTTAGTTTGGACAGGAT-3′	85

**Table 2:** Criteria used for DAI scoring.

DAI Scores			
Scores	Weight Loss (%)	Stool Consistency	Occult/Gross Bleeding
0	None	Normal	Normal
1	1–5		
2	6–10	Loose stool	Occult bleeding
3	11–20		
4	>20	Diarrhea	Gross bleeding

**Table 3:** Criteria used for HIS scoring.

HIS Scores		
Feature Scored	Score	Description
Inflammation severity	0	None
	1	Mild
	2	Moderate
	3	Severe
Inflammation extent	0	None
	1	Mucosa
	2	Mucosa and submucosa
	3	Transmural
Crypt damage	0	None
	1	Basal 1/3 damaged
	2	Basal 2/3 damaged
	3	Crypts lost; surface epithelium present
	4	Crypts and surface epithelium lost
Percent involvement	0	0%
	1	1–25%
	2	26–50%
	3	51–75%
	4	76–100%

**Table 4:** Assay items of clinical chemistry in rat serum.

	Normal	DSS	DSS + A8
TP (g/dL)	6.8	4.8	3.0
ALB (g/dL)	4.6	2.8	1.6
GLU (mg/dL)	205	70	56
BUN (mg/dL)	28.1	17.6	12.6
CRE (mg/dL)	0.5	0.3	0.2
UA (mg/dL)	1.9	4	1.8
Fe (μg/dL)	193	190	88
AST (IU/L)	167	546	470
ALT (IU/L)	42	42	42
ALP (IU/L)	204	30	58
LDH (IU/L)	2963	2870	2030
AMY (IU/L)	1332	836	436
CK (IU/L)	1383	7620	5024
r-GT (IU/L)	<3	<6	<6
ChE (IU/L)	<10	<10	<10
Lip (U/L)	10	8	24
T-CHO (mg/dL)	62	50	18
LDL-C (mg/dL)	5	8	2
HDL-C (mg/dL)	28	16	6

## Data Availability

The data presented in this study are available in this article.

## Supplementary Materials

The following supporting information can be downloaded at https://www.mdpi.com/article/10.3390/biology13110916/s1, Figure S1: The original PCR and the agarose gel electrophoresis assay figures of rectal mRNA expression in rats (Figure 6C). Panels A, B, and C show the results of rat S100A8, S100A9, and β-actin detection, respectively. The leftmost lanes in all images are migrating 100 bp DNA ladder markers. Lanes 2, 3, and 4 from the left show results for the Normal, DSS, and DSS+A8 groups, respectively. The gels were stained with ethidium bromide, and chemiluminescence was detected using a ChemiDoc XRS+ System (Bio-Rad Laboratories, Inc.). Figure S2. The original SDS-PAGE figures regarding fractionation and separation of rectal proteins in UCR group (Figure 3B). The concentration of polyacrylamide gels was 12.5% in the presence of 2-mercaptoethanol. Coomassie Brilliant Blue staining was performed to visualize the protein bands in each gel. Lane M is the molecular weight markers, and lanes 1 to 18 are the eighteen fractions after gel filtration chromatography (see Section 2.7). The protein bands were detected using the ChemiDoc^TM^ XRS Plus Imaging System (Bio-Rad Laboratories, Inc., Hercules, CA, USA). Figure S3. The original Western blotting figures of CA-III and β-action detected in the colon of WT rats and UCR group on Day 10 (Figure 6B). The large colon was divided into three segments (R: rectum, M: middle colon, P: proximal colon), and the proteins extracted from them were subjected to SDS-PAGE (see Section 2.8). After SDS-PAGE, the proteins were transferred to nitrocellulose membranes (0.2 μm pore size) using a Trans-Blot Turbo (Bio-Rad Laboratories, Inc., Hercules, CA, USA). After the membranes were blocked with Blocking One (Nacalai Tesque Co., Ltd., Kyoto, Japan), they were incubated at 4 °C for 1 h with 2 μg/mL of anti-CAIII antibody (upper two panels) or anti-β-actin antibody (lower two panels). The membranes were then washed three times for 5 min with 10 mM Tris-HCl buffer (pH 7.4) and 0.9% NaCl (buffer A), twice with buffer A/0.1% Tween 20, and once with buffer A before being incubated with 2 μg/mL rabbit anti-mouse IgG H&L (HRP) at room temperature for 1 h. After the membranes were washed, antibody-bound proteins were detected using the ChemiDoc^TM^ XRS Plus Imaging System and Clarity Western ECL substrate (Bio-Rad Laboratories, Inc.). Figure S4. The original Western blotting figures of CA-III and β-action detected in MΦ (Figure 7A). The proteins extracted from MΦ stimulated with LPS for 0, 0.5, 1, 2 h were subjected to SDS-PAGE (see Section 2.8). After SDS-PAGE, the proteins were transferred to nitrocellulose membranes (0.2 μm pore size) using a Trans-Blot Turbo (Bio-Rad Laboratories, Inc., Hercules, CA, USA). After the membranes were blocked with Blocking One (Nacalai Tesque Co., Ltd., Kyoto, Japan), they were incubated at 4 °C for 1 h with 2 μg/mL of anti-CAIII antibody (left panel) or anti-β-actin antibody (right panel). The membranes were then washed three times for 5 min with 10 mM Tris-HCl buffer (pH 7.4) and 0.9% NaCl (buffer A), twice with buffer A/0.1% Tween 20, and once with buffer A before being incubated with 2 μg/mL rabbit anti-mouse IgG H&L (HRP) at room temperature for 1 h. After the membranes were washed, antibody-bound proteins were detected using the ChemiDoc^TM^ XRS Plus Imaging System and Clarity Western ECL substrate (Bio-Rad Laboratories, Inc.). Figure S5. The original Western blotting figures of CA-III and β-action detected in MΦ (Figure 8A). The proteins extracted from MΦ incubated with anti-CAIII for 0.5, 1, 2 h were subjected to SDS-PAGE (see Section 2.8). After SDS-PAGE, the proteins were transferred to nitrocellulose membranes (0.2 μm pore size) using a Trans-Blot Turbo (Bio-Rad Laboratories, Inc., Hercules, CA, USA). After the membranes were blocked with Blocking One (Nacalai Tesque Co., Ltd., Kyoto, Japan), they were incubated at 4 °C for 1 h with 2 μg/mL of anti-CAIII antibody (left panel) or anti-β-actin antibody (right panel). The membranes were then washed three times for 5 min with 10 mM Tris-HCl buffer (pH 7.4) and 0.9% NaCl (buffer A), twice with buffer A/0.1% Tween 20, and once with buffer A before being incubated with 2 μg/mL rabbit anti-mouse IgG H&L (HRP) at room temperature for 1 h. After the membranes were washed, antibody-bound proteins were detected using the ChemiDoc^TM^ XRS Plus Imaging System and Clarity Western ECL substrate (Bio-Rad Laboratories, Inc.).
