# Supplementary material for: Intraperitoneal Administration of S100A8 Ameliorates Experimental Acute Colitis in Rats
Source: Biology (Basel). 2024 Nov 11;13(11):916. doi: 10.3390/biology13110916 (PMC11592024; doi:10.3390/biology13110916)
Supplement: Supplementary file 1 [file biology-13-00916-s001.zip › biology-3279716-supplementary.pdf]

Supplementary Materials

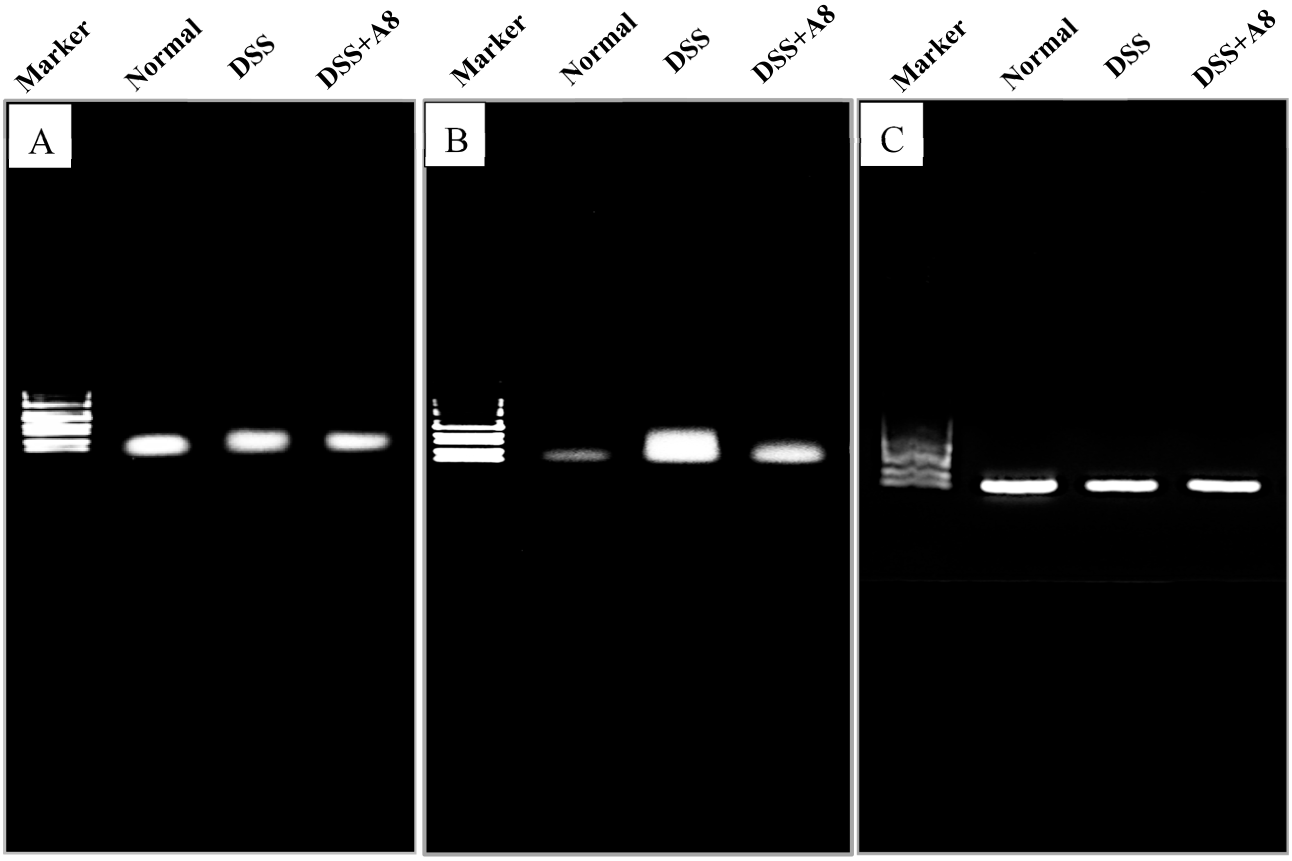

Figure S1. The original PCR and the agarose gel electrophoresis assay figures of rectal mRNA expression in rats (Figure 6C). Panels A, B, and C show the results of rat S100A8, S100A9, and β-actin detection, respectively. The leftmost lanes in all images are migrating 100 bp DNA ladder markers. Lanes 2, 3, and 4 from the left show results for the Normal, DSS, and DSS+A8 groups, respectively. The gels were stained with ethidium bromide, and chemiluminescence was detected using a ChemiDoc XRS+ System (Bio-Rad Laboratories, Inc.).

UCR

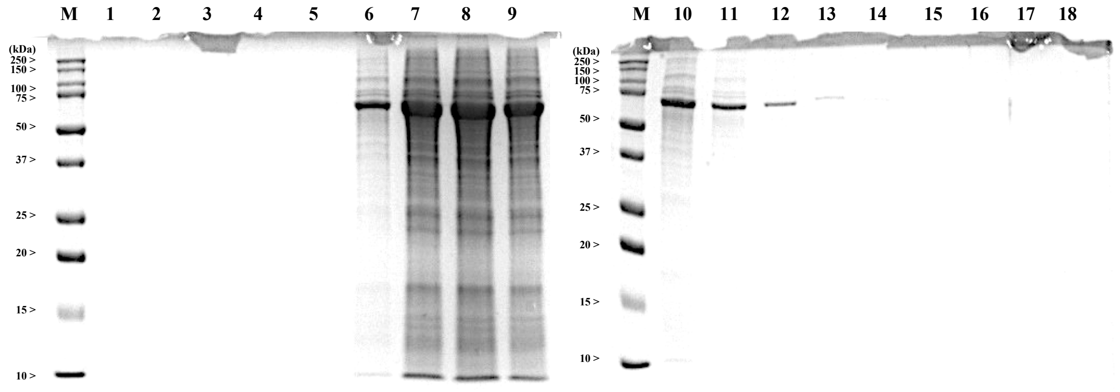

Figure S2. The original SDS-PAGE figures regarding fractionation and separation of rectal proteins in UCR group (Figure 3B). The concentration of polyacrylamide gels was 12.5% in the presence of 2-mercaptoethanol. Coomassie Brilliant Blue staining was performed to visualize the protein bands in each gel. Lane M is the molecular weight markers, and lanes 1 to 18 are the eighteen fractions after gel filtration chromatography (see Section 2.7). The protein bands were detected using the ChemiDoc™ XRS Plus Imaging System (Bio-Rad Laboratories, Inc., Hercules, CA, USA).

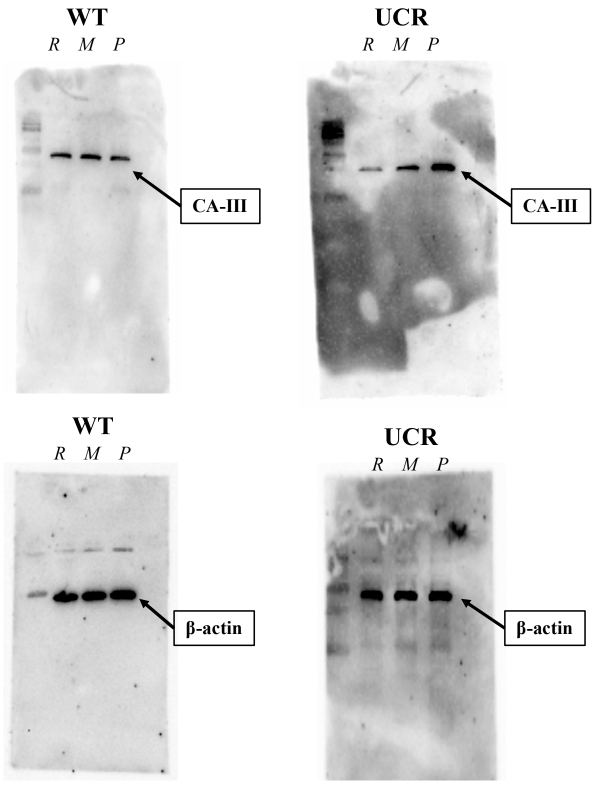

Figure S3. The original Western blotting figures of CA-Ⅲ and β-action detected in the colon of WT rats and UCR group on Day 10 (Figure 6B). The large colon was divided into three segments (R: rectum, M: middle colon, P: proximal colon), and the proteins extracted from them were subjected to SDS-PAGE (see Section 2.8). After SDS-PAGE, the proteins were transferred to nitrocellulose membranes (0.2 µm pore size) using a Trans-Blot Turbo (Bio-Rad Laboratories, Inc., Hercules, CA, USA). After the membranes were blocked with Blocking One (Nacalai Tesque Co., Ltd., Kyoto, Japan), they were incubated at 4 °C for 1 h with 2 µg/mL of anti-CAIII antibody (upper two panels) or anti-β-actin antibody (lower two panels). The membranes were then washed three times for 5 min with 10 mM Tris-HCl buffer (pH 7.4) and 0.9% NaCl (buffer A), twice with buffer A/0.1% Tween 20, and once with buffer A before being incubated with 2 µg/mL rabbit anti-mouse IgG H&L (HRP) at room temperature for 1 h. After the membranes were washed, antibody-bound proteins were detected using the ChemiDoc™ XRS Plus Imaging System and Clarity Western ECL substrate (Bio-Rad Laboratories, Inc.).

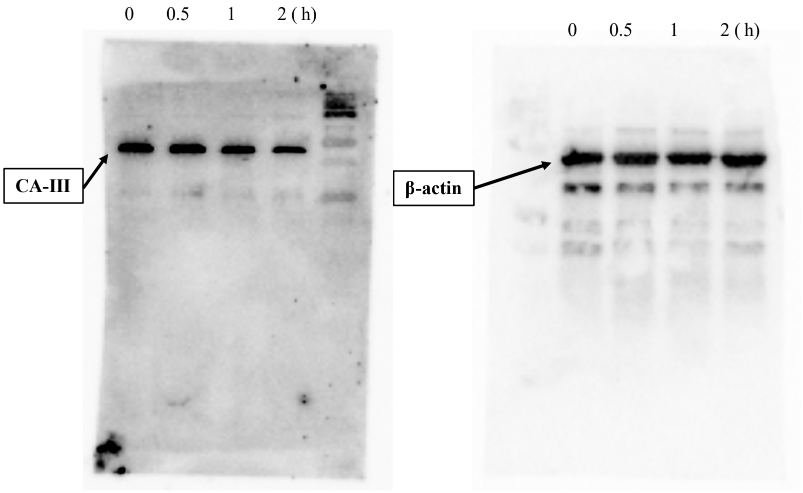

Figure S4. The original Western blotting figures of CA-Ⅲ and β-action detected in MΦ (Figure 7A). The proteins extracted from MΦ stimulated with LPS for 0, 0.5, 1, 2 h were subjected to SDS-PAGE (see Section 2.8). After SDS-PAGE, the proteins were transferred to nitrocellulose membranes (0.2 μm pore size) using a Trans-Blot Turbo (Bio-Rad Laboratories, Inc., Hercules, CA, USA). After the membranes were blocked with Blocking One (Nacalai Tesque Co., Ltd., Kyoto, Japan), they were incubated at 4 °C for 1 h with 2 μg/mL of anti-CAIII antibody (left panel) or anti-β-actin antibody (right panel). The membranes were then washed three times for 5 min with 10 mM Tris-HCl buffer (pH 7.4) and 0.9% NaCl (buffer A), twice with buffer A/0.1% Tween 20, and once with buffer A before being incubated with 2 μg/mL rabbit anti-mouse IgG H&L (HRP) at room temperature for 1 h. After the membranes were washed, antibody-bound proteins were detected using the ChemiDoc™ XRS Plus Imaging System and Clarity Western ECL substrate (Bio-Rad Laboratories, Inc.).

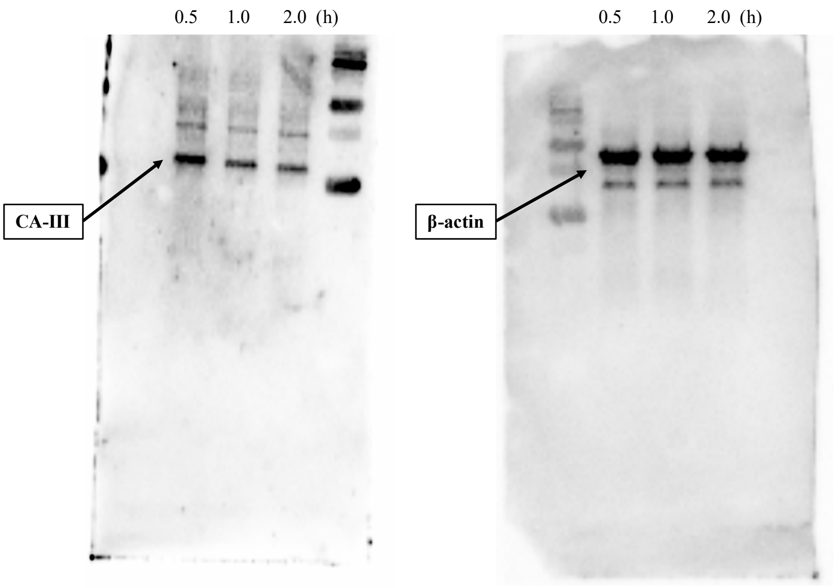

Figure S5. The original Western blotting figures of CA-Ⅲ and β-action detected in MΦ (Figure 8A). The proteins extracted from MΦ incubated with anti-CAIII for 0.5, 1, 2 h were subjected to SDS-PAGE (see Section 2.8). After SDS-PAGE, the proteins were transferred to nitrocellulose membranes (0.2 μm pore size) using a Trans-Blot Turbo (Bio-Rad Laboratories, Inc., Hercules, CA, USA). After the membranes were blocked with Blocking One (Nacalai Tesque Co., Ltd., Kyoto, Japan), they were incubated at 4 °C for 1 h with 2 μg/mL of anti-CAIII antibody (left panel) or anti-β-actin antibody (right panel). The membranes were then washed three times for 5 min with 10 mM Tris-HCl buffer (pH 7.4) and 0.9% NaCl (buffer A), twice with buffer A/0.1% Tween 20, and once with buffer A before being incubated with 2 μg/mL rabbit anti-mouse IgG H&L (HRP) at room temperature for 1 h. After the membranes were washed, antibody-bound proteins were detected using the ChemiDoc™ XRS Plus Imaging System and Clarity Western ECL substrate (Bio-Rad Laboratories, Inc.).
